# The evolution of health policy and systems research in 11 low- and middle-income countries and the role of the Alliance for Health Policy and Systems Research: a bibliometric analysis for 1999–2020

**DOI:** 10.1186/s12961-024-01254-z

**Published:** 2025-01-02

**Authors:** Nino Paichadze, Emma K. Cook, Heather E. Rosen, Sara Kurtovic, Adnan A. Hyder

**Affiliations:** Center on Commercial Determinants of Health and Department of Global Health, Milken Institute School of Public Health, The George Washington University, Washington, DC United States of America

**Keywords:** Bibliometrics, Health policy, Public health systems research, Developing countries

## Abstract

**Objectives:**

Given the rapid growth of the field of health policy and systems research (HPSR), it is important to monitor the research environment, especially the evolution of HPSR research outputs in low- and middle-income countries (LMICs). The objective of this study was to generate quantitative metrics to assess the production of HPSR publications and the role of the Alliance for Health Policy and Systems Research (the Alliance) grant-funded projects in 11 LMICs over the past 20 years.

**Methods:**

We conducted a systematic literature search for HPSR literature from 1999 to 2020 pertaining to 11 target LMIC countries, including grey literature. We analysed the frequency of publications over time, by country and by thematic area. We then used a database of the Alliance’s previous grantees to analyse authorship by Alliance-funded investigators.

**Results:**

HPSR publications across all 11 target countries increased steadily over the past two decades and the rate of publication increased by an average of 34% per year. The majority of HPSR publications during the study period were in health systems (23%) and health workforce (19%) thematic areas. Nineteen per cent of HPSR publications during this time were authored by Alliance-funded investigators. There was extensive heterogeneity between countries both in number of publications and in proportion of publications authored by Alliance-funded investigators.

**Conclusions:**

Growth in the HPSR research environment reflects the expanding prominence of the HPSR field and increased HPSR research capacity in the 11 target countries. Alliance-funded investigators made an important contribution to the growth in HPSR output in these countries.

## Introduction

The field of health policy and system research (HPSR) has grown considerably over the past two decades [1]. Substantial growth has been seen in donor funding, publications and research capacity, globally [2]. Since its inception in 1999, the Alliance for Health Policy and Systems Research (the Alliance) has been promoting the generation and use of HPSR as a means to strengthen health systems in low- and middle-income countries (LMICs) [3]. The Alliance has been an key funder of HPSR research, supporting close to 1600 researchers in 79 LMICs in the last 20 years [4]. During its first 10 years of operation, the Alliance worked specifically to develop the research capacity of younger researchers in LMICs through its grants [5]. In the past decade, the Alliance has broadened its focus, but maintained a strong emphasis on generation of knowledge in HPSR [5].

Given the rapid growth of HPSR, it is important to monitor the research environment, especially the evolution of HPSR research outputs in LMICs. Bibliometrics is “the application of mathematical and statistical methods to books and other media of communication” and can be used to study the productivity of scientific literature in a given topic [6]. Bibliometric analysis is useful for revealing historical trends, evaluating the strengths and weaknesses in the evidence base, and tracing the emergence of new disciplines and thus appropriate for evaluation of HPSR knowledge production [7]. Bibliometric analyses have been used to track literature development in health policy [8], health services research [9] and health systems [10] fields.

A few previous studies have looked at trends in research output in HPSR and structural properties of the HPSR coauthorship network [1, 11–13]. The first bibliometric analysis of HPSR publications found that publications increased from 2003 to 2009, but only 10% of publications were from LMICs and of those publications, only 4% were led by authors from low-income countries [11]. A second analysis covered the longer period 1990–2015, reporting that the pace of output of HPSR papers with a LMIC topic and LMIC lead author was greater than the rate of increase of PubMed publications overall [1]. The coauthorship network analysis from that investigation found that global connectivity and lead authorship of upper middle-income country authors publishing on HPSR was comparable to high-income country authors, while low-income and lower middle-income country authors were lagging behind [12]. Finally, HPSR publications in 47 LMICs were investigated in the period of 2010–2020, where an increasing trend was seen and India, China and Brazil had the highest number of publications [13].

The present study is part of a larger study in which we were engaged by the Alliance to assess the impact and contributions of the Alliance’s HPSR projects in 11 LMICs where the Alliance’s funding was concentrated during the past two decades (1999–2020). For this study, we applied bibliometric methods to evaluate HPSR scientific output over time. The overall goal of the study was to generate quantitative metrics to assess the evolution of HPSR publications in the 11 target countries. We also wanted to assess the role of the Alliance’s grant support in HPSR growth over time. Evaluation of HPSR evidence production, especially in LMIC settings, is crucial to understand the growth of the discipline, assess gaps and plan for future investment.

## Methods

### Systematic review

We developed a search strategy in consultation with a library information specialist at the George Washington University. We used broad keywords to ensure the inclusion of the maximum number of publications. Our search terms included the names of the 11 target countries (“Brazil” OR “China” OR “Ghana” OR “India” OR “Lebanon” OR “Mexico” OR “Nigeria” OR “Pakistan” OR “South Africa” OR “Uganda” OR “Vietnam”) in combination with three terms for HPSR (“health policy and systems research” OR “health systems” OR “health policy research”). All searches were conducted in July 2020. We ran searches in PubMed, Global Health and Global Index Medicus for the dates 1 January 1999 to 1 May 2020; the end date was selected to allow us to capture as many publications as possible. For Google Scholar, studies recommend using the first 200–300 most relevant search results [14, 15]. We retrieved a large number of records from Google Scholar so we decided to screen the first 1500 records sorted by relevance, and include in the analysis the first 999 most relevant records. The search results were transferred to Covidence [16], a well-recognized web-based platform used for the management of search results and data extraction for systematic and scoping literature reviews.

Title and abstract screening were conducted independently by three team members (EKC, SK and HER) with a fourth team member (NP) reviewing and resolving conflicts. Random checks in which the three reviewers independently categorized the same set of records were conducted to ensure interrater reliability. Publications were included if an abstract was available in English, if the date of publication was between 1 January 1999 and 1 May 2020, if the content was one of the 11 target countries and if the content was HPSR. HPSR was categorized into eight thematic areas: governance and leadership, health policy, health workforce, health financing, health information, medicines and supplies, health systems and health policy research. These categories were loosely based on the WHO’s six building blocks of health systems [17]. We made a distinction between HPSR content (which was included) and health services research (HSR) content (which was excluded). We categorized HSR according to the framework from Sheikh et al. [18] to include studies focused on the effectiveness of specific health interventions, clinical service delivery, translation of research into clinical practice and implementation science. This allowed the setting of manageable boundaries for our study and was consistent with framing HPSR as a field shaped by questions rather than by specific methodologies [18].

Records were excluded for the following reasons consistent with the outlined inclusion criteria: the focus of the publication was HSR and not HPSR, the title and abstract were in a language other than English, the publication was not related to HPSR, the publication was not related to any of the 11 target countries, no abstract was available for the publication or the date of publication was outside of the study period. During the title and abstract screening, data were extracted from each publication on the thematic area, focal country or countries, year of publication and authors.

### Alliance-funded investigators

To analyse the impact of Alliance funding on the research environment, we needed to access the names of researchers funded by Alliance grants. The Alliance provided a complete database of their grants in the 11 target countries in the years 1999–2020, which included 247 grants and 343 primary investigators and co-investigators (hereafter referred to collectively as investigators). Unfortunately, 18 grants were missing the names of all investigators and one investigator name was missing the given name; thus, we approached the analysis with a list of 229 grants and 342 investigators. The number of grants and investigators in each country varied.

### Bibliometric analysis

We conducted data analyses using Microsoft Excel and Stata statistical software, release 16 [19]. The records identified by the systematic review were analysed to assess: (1) publications over time, (2) publications by thematic area and (3) publications by country. We then separated the records into publications authored by Alliance-funded investigators and authors not funded by the Alliance. We looked again at publications over time, by thematic area and by country.

## Results

### Systematic review

**Fig. 1:**
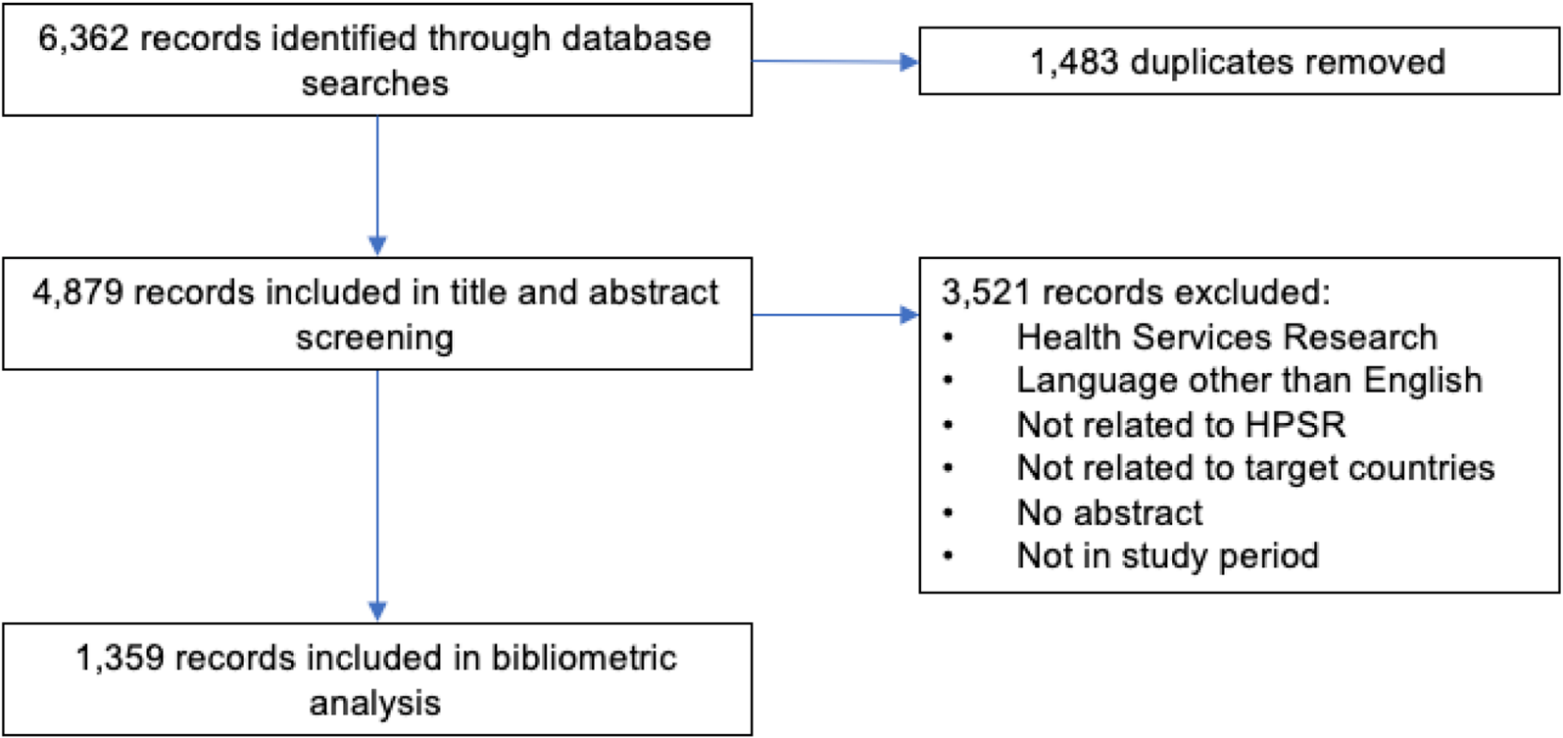
Flowchart of the systematic review process

The database searches returned 6362 records with 1483 duplicates (23%) which were removed, resulting in a final set of 4879 records that were included in the next stage (Fig. 1). After title and abstract screening, 3521 (72%) records were excluded from further analysis. The resulting 1359 records remaining after the screening were included in the bibliometric analysis.

### Bibliometric analysis

**Fig. 2:**
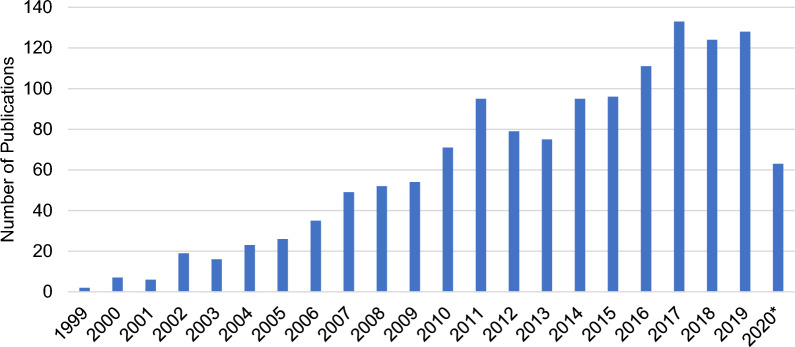
HPSR publications per year, 1999–2020. *1 January–1 May 2020

HPSR publications across the 11 target countries increased steadily from 1999 to 2020 with a low of 2 publications per year in 1999 and a high of 133 publications per year in 2017 (Fig. 2). The average increase in publications per year from 1999 to 2019 (the last full year) was 34%.

Across the 11 target countries, the health systems and health workforce thematic areas had the most publications in the study period, with 308 (23%) and 258 (19%), respectively (Table 1). There were few publications in the health information (*n* = 44, 3%), health policy research (*n* = 89, 7%), and medicines and supplies (*n* = 98, 7%) thematic areas.

**Table 1:** HPSR publications by thematic area, 1999–2020

Thematic area	Number	Percentage
Health systems	308	22.7
Health workforce	258	19.0
Health financing	198	14.6
Health policy	189	13.9
Governance and leadership	175	12.9
Medicines and supplies	98	7.2
Health policy research	89	6.5
Health information	44	3.2

Across the past two decades, there has been a wide range in the number of HPSR publications by country, with Brazil having the most publications (*n* = 391 publications, 26%) and Lebanon having the fewest publications (*n* = 15, 1%; Table 2). Along with Brazil, South Africa and India also had high numbers of publications. South Africa (3.89), Uganda (3.12) and Ghana (2.89) had the highest rates of publications per million population.

**Table 2:** HPSR publications by country and per million population, 1999–2020

Country	Number of publications*	Percentage of publications	Publications per million population [20]
Brazil	391	25.5	1.82
South Africa	231	15.1	3.89
India	222	14.5	0.16
Uganda	143	9.3	3.12
Mexico	127	8.3	1.00
China	112	7.3	0.08
Nigeria	100	6.5	0.47
Ghana	95	6.2	2.89
Pakistan	76	5.0	0.33
Vietnam	22	1.4	0.23
Lebanon	15	1.0	2.68

^*^Sum of individual country values is more than total publications because some publications featured multiple countries

### Authorship by alliance-funded investigators

Of the 1359 HPSR publications analysed, 261 (19%) were authored by Alliance-funded investigators (Table 3). These 261 publications were authored by 123 unique individuals out of 342 total Alliance-funded investigators meaning that 36% of Alliance-funded investigators authored a publication. Governance and leadership (*n* = 42, 24%), health policy research (*n* = 21, 24%) and health financing (*n* = 46, 23%) were the thematic areas with highest percentage of publications authored by Alliance-funded investigators (Table 3). The health information thematic area had the lowest percentage of publications by Alliance-funded investigators at only 7% (*n* = 3).

**Table 3:** HPSR publications by thematic area and funding, 1999–2020

Thematic area	Alliance-funded publications	Alliance-funded percentage of publications	Non-alliance funded publications	Non-alliance-funded percentage of publications	Total publications
Health systems	43	14.0	265	86.0	308
Health workforce	51	19.8	207	80.2	258
Health financing	46	23.2	152	76.8	198
Health policy	39	20.6	150	79.4	189
Governance and leadership	42	24.0	133	76.0	175
Medicines and supplies	16	16.3	82	83.7	98
Health policy research	21	23.6	68	76.4	89
Health information	3	6.8	41	93.2	44

Lebanon, India and Nigeria had the highest percentages of HPSR publications authored by Alliance-funded investigators with 53% (*n* = 8), 32% (*n* = 70) and 29% (*n* = 29), respectively (Table 4). Brazil had the lowest percentage of Alliance-funded investigator authorship at 2% (*n* = 9).

**Table 4:** HPSR publications by country and funding, 1999–2020

Country	Alliance-funded publications*	Alliance-funded percentage of publications	Non-alliance-funded publications*	Non-alliance-funded percentage of publications	Total publications*
Brazil	9	2.3	382	97.7	391
South Africa	64	27.7	167	72.3	231
India	70	31.5	152	68.5	222
Uganda	38	26.6	105	73.4	143
Mexico	19	15.0	108	85.0	127
China	15	13.4	97	86.6	112
Nigeria	29	29.0	71	71.0	100
Ghana	26	27.4	69	72.6	95
Pakistan	17	22.4	59	77.6	76
Vietnam	2	9.1	20	90.9	22
Lebanon	8	53.3	7	46.7	15

^*^Sum of individual country values is more than total publications because some publications featured multiple countries

## Discussion

In this analysis, we assessed HPSR publications across 11 LMICs over the past two decades to understand the evolution of HPSR in these countries and the role played by the Alliance. We found that, across the 11 target countries, HPSR publications have steadily and substantially increased since 1999 at an average rate of 34% per year. This rate is higher than the estimated growth rate of all science publications of 3% per year [21], indicating that HPSR publications in these 11 countries are increasing at a faster pace than global scientific output in general.

Overall, HPSR publications in the 11 countries were heavily concentrated in two thematic areas: health systems and health workforce. Adam et al. similarly found that human resources was one of the most popular topics in HPSR publications related to LMICs between 2003 and 2009 [11]. We found the fewest publications in the area of health information which includes health information and surveillance systems, standardized tools and instruments, and international health statistics [17]. This indicates an area of HPSR that may need increased funding and attention.

Output of HPSR publications varied considerably between countries from 1999 to 2020. There was a relatively high number of HPSR publications in Brazil, South Africa and India while Lebanon, Vietnam, Pakistan and Ghana had low numbers of HPSR publications. While we do not have data on the total number of HPSR investigators in the 11 countries, looking at publication rates by million population can provide interesting insight. Lebanon and Ghana, two countries with low HPSR publication output overall, have high rates of publications per million population. Alternatively, India had high publication output compared with the other countries, but one of the lowest rates of publications per million population. This potentially indicates that some of the trends seen in publication at a country level are driven by other factors that are not measured in this analysis.

When we analysed publications by author name, we found that 19% of all HPSR publications during the time period in these countries were authored by Alliance-funded investigators. We cannot say for sure whether an Alliance-funded project was the topic of these papers or whether Alliance funding had any impact on the publication process. Nonetheless, it is clear that researchers funded by the Alliance were productive in terms of evidence generation, fulfilling a primary objective of Alliance support.

Lebanon had a high share (over 50%) of HPSR publications authored by Alliance-funded investigators, suggesting that the Alliance played a substantial role in research production in this country. On the other hand, Brazil had more HPSR publications than any other country and Alliance-funded investigators represented a small proportion of the HPSR output at 2%. It seems that the research environment in each country was at a different stage of development.

The main strength of this analysis is the rigorous methodology. After conducting the literature searches, we took the further step of screening our search results by title and abstract which ensured that the maximum number of relevant publications were included in the review, while omitting publications which did not focus on HPSR. The thorough screening process allowed us to use broad search terms and cast a wide net for literature. The inclusion of grey literature via Google Scholar also added depth to our analysis beyond previous HPSR bibliometric analyses.

This bibliometric analysis had several limitations. First, the exclusion of publications without abstracts in English likely resulted in the omission of relevant publications written in other languages. Publishing in the language spoken by the country’s researchers and policy-makers is a good strategy to promote dissemination and uptake of research. This was primary an issue for Mexico and Brazil so it may have reduced the validity of our findings from those two countries. In addition, the use of the health systems building blocks to categorize publications thematically presents a limitation in that these categories are largely overlapping and inconsistent in their definitions [1]. Also, the author-level analyses were based on the database that we received from the Alliance and relies on the accuracy of that data. Further, because articles are often published months or years after a project ends, it is likely that our analysis missed some Alliance-funded grant-related publications. Finally, potential transcription errors for author names with accents could have impeded identification of Alliance-funded investigators in this analysis, especially relevant for authors in Mexico and Brazil.

## Conclusions

Over the past two decades, the HPSR research environment has expanded considerably in the 11 target countries where the Alliance has been highly involved. Our analysis of HPSR publications by Alliance-funded investigators suggests that the Alliance played a key role in building research capacity and output in these countries.

## Data Availability

The data from this study are available from the authors upon reasonable request.
